# Portal hypertensive gastropathy as a prognostic index in patients with liver cirrhosis

**DOI:** 10.1186/s12876-016-0508-2

**Published:** 2016-08-12

**Authors:** Chang Seok Bang, Hyo Sun Kim, Ki Tae Suk, Sung Eun Kim, Ji Won Park, Seung Ha Park, Hyoung Su Kim, Myoung Kuk Jang, Sang Hoon Park, Myung Seok Lee, Choong Kee Park, Dong Joon Kim

**Affiliations:** 1 Department of Internal Medicine, Hallym University College of Medicine, Chuncheon, Gangwon-do 24253 South Korea; 2 Department of Internal Medicine, Inje University Haeundae-Paik Hospital, Busan, South Korea

**Keywords:** Cirrhosis, Portal hypertension, Portal hypertensive gastropathy, Hepatocellular carcinoma

## Abstract

**Background:**

Portal hypertensive gastropathy (PHG) is a frequently overlooked complication of liver cirrhosis (LC). The clinical implications of PHG as a prognostic factor of LC or a predictive factor for the development of hepatocellular carcinoma (HCC) have not been established. The aim of this study was to assess the clinical significance of PHG in patients with LC.

**Methods:**

Patients with LC were prospectively enrolled and followed in a single tertiary hospital in the Republic of Korea. Baseline hepatic vein pressure gradient (HVPG) was measured, and esophagogastroduodenoscopy (EGD) was performed. The associations of PHG with HVPG, survival and the development of HCC were evaluated.

**Results:**

A total of 587 patients were enrolled. The mortality rate was 20.3 % (*n* = 119), and HCC developed in 9.2 % (*n* = 54) during the follow-up period (32.6 ± 27.8 months). The grade of PHG was well correlated with HVPG (no PGH: median 9.2 [IQR: 7.2–16.7], mild PHG: 14.6 [10.1–19.3], and severe PHG: 17.3 [12.3–21.5], *P* < 0.001), as well as with Child-Pugh class, MELD score or survival. However, it was not associated with the development of HCC. The grade of PHG (HR 3.29, 95 % CI: 1.12–9.63, severe vs. no PHG) and Child-Pugh class (HR 3.53, 95 % CI: 1.79–6.97, Child C vs A) showed significant associations with mortality.

**Conclusion:**

PHG was well correlated with portal hypertension and could be used as a prognostic factor for LC but not for the prediction of HCC.

**Electronic supplementary material:**

The online version of this article (doi:10.1186/s12876-016-0508-2) contains supplementary material, which is available to authorized users.

## Background

Portal hypertension is a complication of liver cirrhosis (LC) and the main pathophysiologic mechanism that potentiates various adverse gastrointestinal consequences, including esophageal or gastric varices, gastropathy, and enteropathy [1, 2].

Portal hypertensive gastropathy (PHG) is a frequently overlooked complication in patients with LC. More attention has been focused on the detection or evaluation of esophageal or gastric varices by endoscopists. This complex secondary change in the gastric mucosa resulting from portal hypertension is a potential cause of acute or chronic hemorrhage [3]. It can also be severe and fatal, although less frequently than variceal hemorrhage [4].

In addition to the potential hemorrhagic focus, the clinical implications of PHG have not been well established. Previous studies have also shown conflicting results regarding the correlation between PHG and the severity of liver disease [5–13]. The aim of this study was to evaluate the clinical implications of PHG as a prognostic factor of LC or a predictive factor for the development of hepatocellular carcinoma (HCC) in patients with LC.

## Methods

### Patients

Patients with chronic liver disease were prospectively enrolled and followed in a single tertiary hospital in the Republic of Korea. Baseline hepatic vein pressure gradient (HVPG) was measured, and esophagogastroduodenoscopy (EGD) was performed in all consecutive patients for the detection or evaluation of the severity of PHG. Both procedures were performed consecutively and the time interval between 2 procedures was minimal. Patients without LC or with incomplete data were excluded from this study. The clinical and endoscopic characteristics of patients with LC were reviewed and analyzed. Data were recorded for the following variables: sex, age, the etiology of LC, endoscopic findings, laboratory findings, and HVPG. Laboratory findings including Child-Pugh classification and Model for End-stage Liver disease (MELD) score were assessed based on hospitalization day.

### Differentiation of etiology

The differentiation between LC and chronic hepatitis relied on clinical, laboratory, radiologic and histologic information. The final determination of LC was made by two hepatologists (K.T.S and D.J.K).

In terms of the etiology, chronic hepatitis B was defined as positive for hepatitis B surface antigen (HBsAg) with abnormal levels of aspartate transaminase (AST) / alanine transaminase (ALT) for a period longer than 6 months. Chronic hepatitis C was defined as positivity for hepatitis C antibodies (Anti-HCV) and serum RNA (HCV-RNA) with abnormal levels of AST/ALT for a period longer than 6 months. Determination of alcoholic hepatitis used history of alcohol abuse (>40 g/day for men, >20 g/day for women) [14, 15], physical findings (delirium tremens or alcohol withdrawal seizure), laboratory tests (AST/ALT >2, elevated level of gamma glutamyl transpeptidase, or enlarged mean corpuscular volume), or liver biopsy (steatosis, hepatocyte ballooning, Mallory-Denk bodies, megamitochondria, canalicular and/or lobular bilirubinostasis, or polymorphonuclear neutrophil infiltration), after excluding other potential etiologies. The determination of non-alcoholic steatohepatitis relied on history (exclusion of significant alcohol consumption), laboratory tests (AST or ALT elevation), imaging modalities (hepatic steatosis), or liver biopsy (macrovesicular fatty changes, hepatocyte ballooning, or inflammatory cell infiltrate), after excluding other potential etiologies.

### Endoscopy and HVPG measurement

PHG was evaluated by EGD performed by 6 experienced endoscopists (>6000 cases of endoscopy). The diagnosis and determination of degree were based on the Baveno III scoring system [16]. To minimize the inter-observer variability, all of the endoscopic data and diagnoses were reviewed by 6 experienced endoscopists. Cases of disagreement were discussed and resolved by consensus, according to the Baveno III scoring system. To exclude single gastric antral vascular ectasia cases, not relevant to portal hypertension, authors categorized enrolled population according to the presence of PHG or not (binary criteria), in addition to the Baveno III scoring system for PHG, and six experienced endoscopists made decisions by consensus.

HVPG was measured with transjugular access under fluoroscopic guidance by 2 experienced hepatologists (K.T.S and S.H.P >300 cases of HVPG measurements). A catheter was placed into one of the hepatic vein branches, and the pressure was measured three times using the ballooning and deballooning method of the hepatic vein. The average number was recorded and decided upon as the patient’s HVPG. Informed consent was obtained. Vital signs were continuously monitored, and the patients were observed carefully to detect the development of serious complications during and after the measurement of HVPG.

### Statistical analysis

Continuous variables are expressed as the medians and interquartile ranges (IQRs) because they were not normally distributed. Categorical variables are expressed as numbers and percentages. The Mann-Whitney test and Fisher’s exact test were used to compare two variables. The Kruskal-Wallis test and Fisher’s exact test were used to evaluate the three study arms. Post hoc analysis was performed using Bonferroni’s correction. Survival analysis, including the development of HCC, was performed using the Kaplan-Meier method and the log rank test. The Cox proportional hazard model was applied for the detection of associated factors for survival and the development of HCC. A *P* value < 0.05 (2-tailed) was adopted as the threshold of statistical significance for all of the tests. The analysis was performed using SPSS software, version 21.0 (SPSS Inc., Chicago, IL, USA). All of the authors had access to the study data and reviewed and approved the final manuscript.

## Results

### Characteristic of patients

**Fig. 1:**
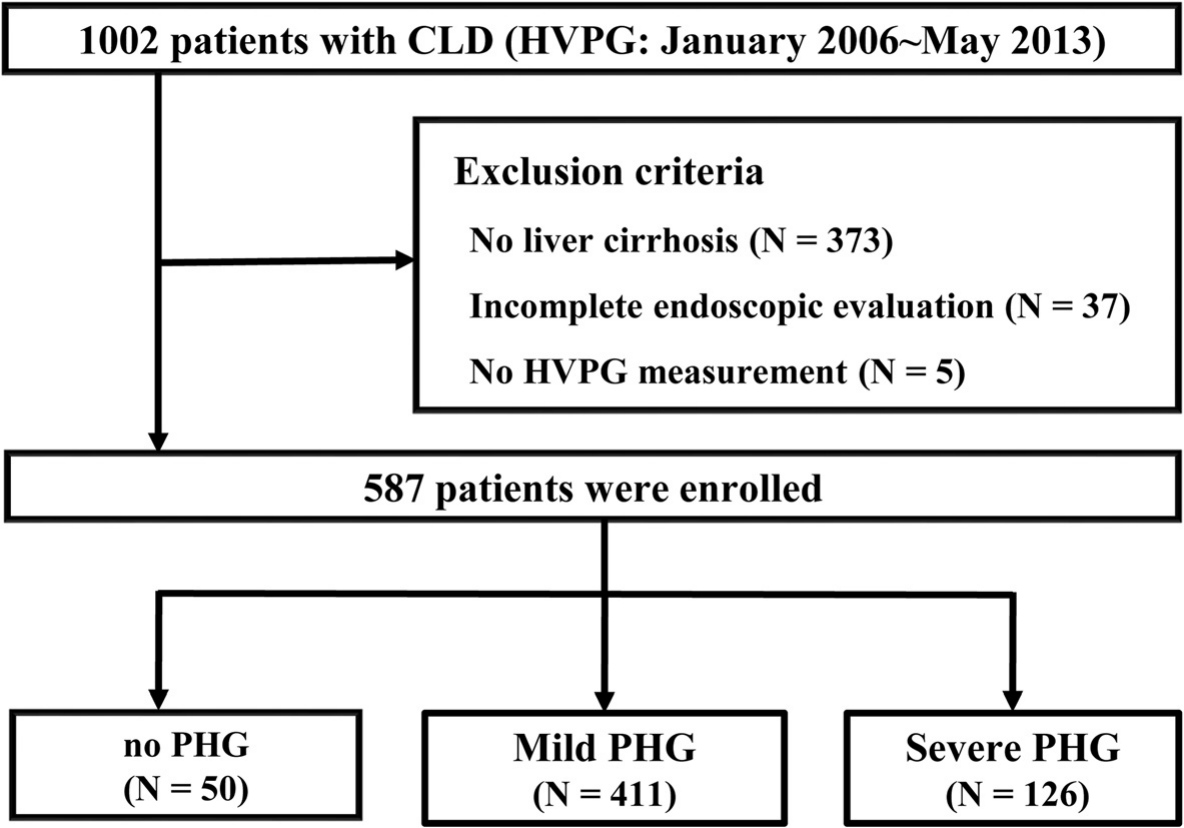
Flow diagram of study design

Between January 2006 and May 2013, 1002 patients were initially enrolled. Patients without LC or with incomplete data were excluded from this study. The number of excluded cases from each category was as follows: no LC (*n* = 373), incomplete endoscopic evaluation (*n* = 37), and incomplete HVPG measurement (*n* = 5). Finally, 587 patients were included in the analysis of this study (Fig. 1).

The clinical characteristics of these patients are summarized in Table 1. The median age was 51 years old (IQR: 45–59) in the total population. Male predominance was observed in the collected data for 78.7 % of the total patients. In terms of the etiology, alcohol abuse was the most frequent cause of LC (69.8 %), followed by hepatitis B virus (HBV) infection (24.2 %), hepatitis C virus (HCV) infection (5.8 %), and non-alcoholic causes (0.2 %).

**Table 1:** Clinical characteristics of total patients

Characteristics, N (%)	Total (*N* = 587)
Age (years), Median (Interquartile range)	51 (45–59)
Sex	
Male	462 (78.7 %)
Female	125 (21.3 %)
Etiology	
Alcohol	410 (69.8 %)
HBV	142 (24.2 %)
HCV	34 (5.8 %)
Non-alcoholic	1 (0.2 %)
PHG	
No PHG	50 (8.5 %)
Mild PHG	411 (70 %)
Severe PHG	126 (21.5 %)
Child-Pugh classification	
Class A	283 (48.2 %)
Class B	234 (39.9 %)
Class C	70 (11.9 %)
HVPG (mmHg), Median (Interquartile range)	14.8 (10–20)
MELD score	9.3 (6.0–13.0)
Albumin (g/dL)	3.3 (2.8–3.8)
Total bilirubin (mg/dL)	1.4 (0.8–2.7)
Platelet count (x 10^3^/mL)	105 (74–160)
Prothrombin time (INR)	1.26 (1.11–1.44)
Follow-up duration (months), Mean ± SD	32.59 ± 27.77
Survival	468 (79.7 %)
HCC	54 (9.2 %)

*N* number, *HBV* hepatitis B virus, *HCV* hepatitis C virus, *PHG* portal hypertensive gastropathy, *HVPG* hepatic vein pressure gradient, *MELD* model for end-stage liver disease, *INR* international normalized ratio, *SD* standard deviation, *HCC* hepatocellular carcinoma

Regarding the severity of LC, approximately half of the patients were included in Child-Pugh classification A (48.2 %), followed in order by B (39.9 %) and C (11.9 %). The median value of HVPG was 14.8 (IQR: 10–20), and the MELD score was 9.3 (IQR: 5.7–13.0).

PHG was detected in 91.5 % of the patients. Among the patients with PHG, the severe category constituted 21.5 %, and 70 % of the patients showed mild PHG. Patients with only gastric antral vascular ectasia was 3 (0.51 %). During the follow-up period (32.59 ± 27.77 months), the overall survival rate was 79.7 %, and 9.2 % of the patients developed HCC. The proportion patients lost to follow-up was 12.8 % (*n* = 75).

There were no serious complications during or after HVPG measurements. Transient ventricular premature contraction was frequently noted when the tip of the measurement catheter passed the right atrium. Cardiac rhythm recovered without any treatment after a few seconds.

#### Univariate analysis for PHG

The univariate analyses for PHG in LC are listed in Tables 2 and 3. There were significant differences in the distributions of Child-Pugh classification, HVPG, and MELD score, as well as laboratory parameters, between patients with and without PHG (Table 2). In the analysis stratified by the severity of PHG (no PHG vs mild PHG vs severe PHG), this finding was consistent (Table 3).

**Table 2:** Univariable analysis for PHG in patients with liver cirrhosis

Variables, N (%)	No PHG	PHG	*P* value
	*N* = 50 (8.5 %)	*N* = 537 (91.5 %)	
Age (years), Median (Interquartile range)	52 (43–60.25)	51 (45–59)	0.64
Sex			0.002
Male	30 (60 %)	432 (80.4 %)	
Female	20 (40 %)	105 (19.6 %)	
Etiology			<0.001
Alcohol	20 (40 %)	390 (72.6 %)	
HBV	26 (52 %)	116 (21.6 %)	
HCV	4 (8 %)	30 (5.6 %)	
Non-alcoholic	0 (0 %)	1 (0.2 %)	
Child-Pugh classification			<0.001
Class A	37 (74 %)	246 (45.8 %)	
Class B	12 (24 %)	222 (41.3 %)	
Class C	1 (2 %)	69 (12.8 %)	
HVPG (mmHg), Median (Interquartile range)	9.2 (7.23–16.73)	15 (11–20)	<0.001
MELD score	6.00 (6.00–8.07)	9.83 (6.00–13.58)	<0.001
Albumin (g/dL)	3.7 (3.0–4.1)	3.2 (2.8–3.7)	0.001
Total bilirubin (mg/dL)	0.9 (0.6–1.2)	1.5 (0.9–2.9)	<0.001
Platelet count (x 10^3^/mL)	141.5 (94.8–214.3)	104 (71–157)	0.001
Prothrombin time (INR)	1.12 (1.04–1.32)	1.28 (1.12–1.45)	<0.001
Survival	46 (92 %)	422 (78.6 %)	0.004
HCC	5 (10 %)	49 (9.1 %)	0.33

*N* number, *HBV* hepatitis B virus, *HCV* hepatitis C virus, *PHG* portal hypertensive gastropathy, *HVPG* hepatic vein pressure gradient, *MELD* model for end-stage liver disease, *INR* international normalized ratio, *SD* standard deviation, *HCC* hepatocellular carcinoma

**Table 3:** Univariable analysis for PHG in patients with liver cirrhosis

Variables, N (%)	No PHG	Mild PHG	Severe PHG	*P* value
	*N* = 50 (8.5 %)	*N* = 411 (70 %)	*N* = 126 (21.5 %)	
Age (years), Median (Interquartile range)	52 (43–60.25)	52 (46–60)	51 (45–56)	0.24
Sex				0.001
Male	30 (60 %)	324 (78.8 %)	108 (85.7 %)	
Female	20 (40 %)	87 (21.2 %)	18 (14.3 %)	
Etiology				<0.001
Alcohol	20 (40 %)	284 (69.1 %)	106 (84.1 %)	
HBV	26 (52 %)	99 (24.1 %)	17 (13.5 %)	
HCV	4 (8 %)	27 (6.6 %)	3 (2.4 %)	
Non-alcoholic	0 (0 %)	1 (0.2 %)	1 (0 %)	
Child-Pugh classification				<0.001
Class A	37 (74 %)	197 (47.9 %)	49 (38.9 %)	
Class B	12 (24 %)	169 (41.1 %)	53 (42.1 %)	
Class C	1 (2 %)	45 (10.9 %)	24 (19 %)	
HVPG (mmHg), Median (Interquartile range)	9.2 (7.2–16.7)	14.6 (10.1–19.3)	17.3 (12.3–21.5)	<0.001
MELD score	6.00 (6.00–8.07)	9.41 (6.00–12.85)	10.64 (6.94–14.73)	<0.001
Albumin (g/dL)	3.7 (3.0–4.1)	3.3 (2.8–3.8)	3.2 (2.8–3.6)	0.001
Total bilirubin (mg/dL)	0.9 (0.6–1.2)	1.4 (0.8–2.5)	1.9 (1.0–4.3)	<0.001
Platelet count (x 10^3^/mL)	141.5 (94.8–214.3)	103 (75–156)	104 (66.8–167)	0.004
Prothrombin time (INR)	1.12 (1.04–1.32)	1.26 (1.11–1.44)	1.29 (1.15–1.49)	<0.001
Survival	46 (92 %)	327 (79.6 %)	95 (75.4 %)	
HCC	5 (10 %)	43 (10.5 %)	6 (4.8 %)	

HVPG, Severe PHG vs Mild PHG: *P* = 0.001, Severe PHG vs No PHG: *P* < 0.001, Mild PHG vs No PHG: *P* < 0.001

MELD score, Severe PHG vs Mild PHG: *P* = 0.015, Severe PHG vs No PHG: *P* < 0.001, Mild PHG vs No PHG: *P* < 0.001

Albumin, Severe PHG vs Mild PHG: *P* = 0.18, Severe PHG vs No PHG: *P* < 0.001, Mild PHG vs No PHG: *P* = 0.002

Total bilirubin, Severe PHG vs Mild PHG: *P* < 0.001, Severe PHG vs No PHG: *P* < 0.001, Mild PHG vs No PHG: *P* < 0.001

Platelet, Severe PHG vs Mild PHG: *P* = 0.49, Severe PHG vs No PHG: *P* = 0.004, Mild PHG vs No PHG: *P* = 0.001

INR, Severe PHG vs Mild PHG: *P* = 0.14, Severe PHG vs No PHG: *P* < 0.001, Mild PHG vs No PHG: *P* < 0.001

Survival, Severe PHG vs Mild PHG: *P* = 0.08, Severe PHG vs No PHG: *P* < 0.001, Mild PHG vs No PHG: *P* = 0.008

HCC, severe PHG vs Mild PHG: *P* = 0.63, Severe PHG vs No PHG: *P* = 0.34, Mild PHG vs No PHG: *P* = 0.32

*N* number, *HBV* hepatitis B virus, *HCV* hepatitis C virus, *PHG* portal hypertensive gastropathy, *HVPG* hepatic vein pressure gradient, *MELD* model for end-stage liver disease, *INR* international normalized ratio, *SD* standard deviation, *HCC* hepatocellular carcinoma

**Fig. 2:**
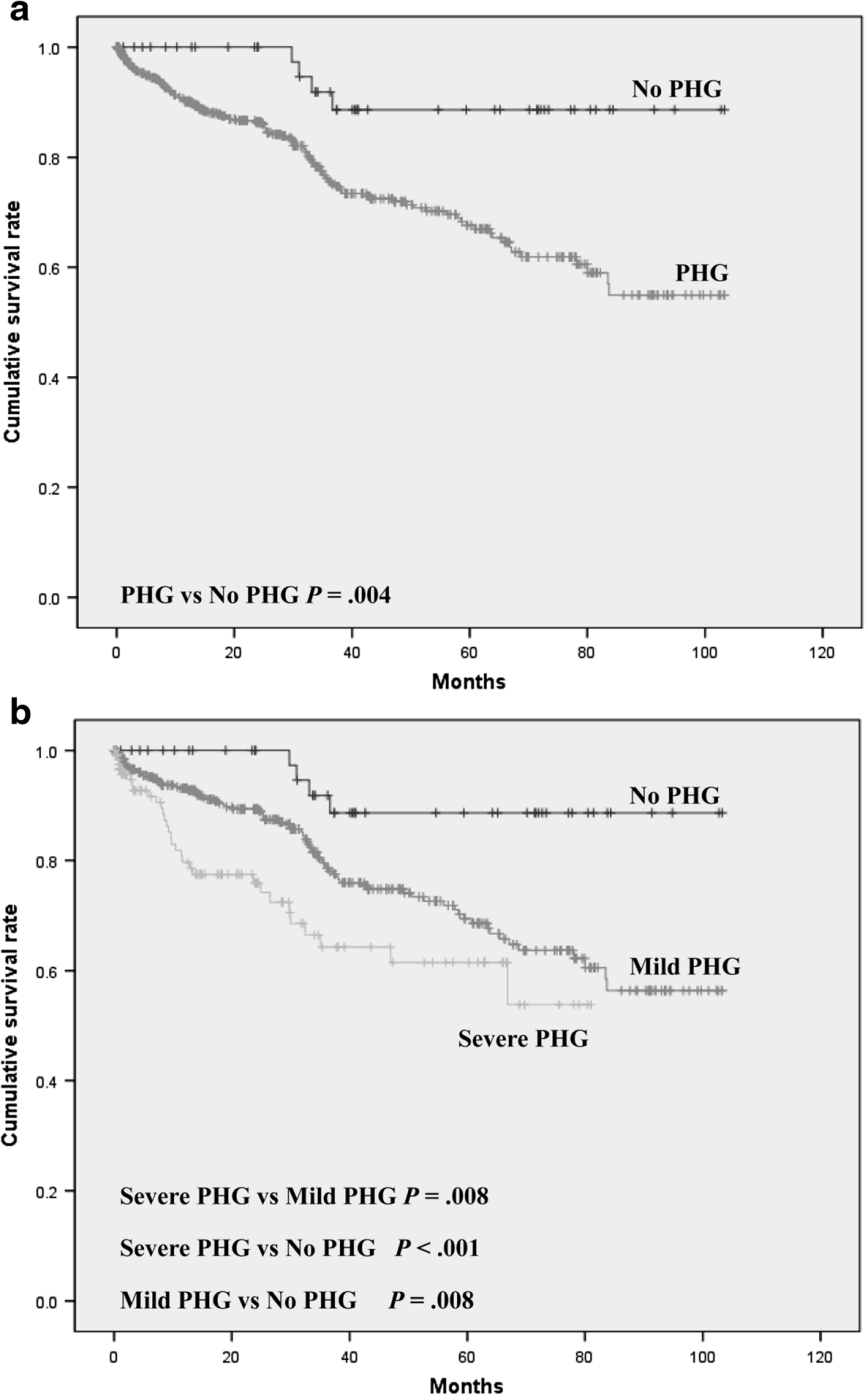
Survival curve according to the presence of PHG (**a**) and PHG grade (**b**). PHG, portal hypertensive gastropathy

In the analysis of survival, patients without PHG showed a higher survival rate than patients with PHG (92 % vs 78.6 %, 95.33 ± 3.78 vs 74.53 ± 2.21 months, *P* = 0.004) (Table 2 and Fig. 2a). This finding was also consistent with the analysis stratified by the severity of PHG (severe PHG vs mild PHG, *P* = 0.008; severe PHG vs no PHG, *P* < 0.001; mild PHG vs no PHG, *P* = 0.008) (Table 3, Fig. 2b). The detailed survival months and survival rates are reported in Table 4.

**Table 4:** Survival rate according to the PHG grade

PHG grade	Mean survival (months), Mean ± SD	Survival rate			Overall mortality (%)
		1 year (%)	3 years (%)	7 years (%)	
No PHG	95.33 ± 3.78	100	88.6	88.6	8
Overall PHG	74.53 ± 2.21	89.9	75.5	54.9	21.4
Mild PHG	76.68 ± 2.40	92.8	78.5	56.4	20.4
Severe PHG	55.48 ± 2.05	79.7	64.3	53.8 (5 years)	24.6

*N* number, *PHG* portal hypertensive gastropathy, *SD* standard deviation

For the development of HCC, there was no significant difference between patients with or without PHG (9.1 % vs 10 %, *P* = 0.33) (Table 2). This finding was also consistent with the analysis stratified by the severity of PHG (severe PHG vs mild PHG: *P* = 0.63; severe PHG vs no PHG: *P* = 0.34; mild PHG vs no PHG: *P* = 0.32) (Table 3).

**Fig. 3:**
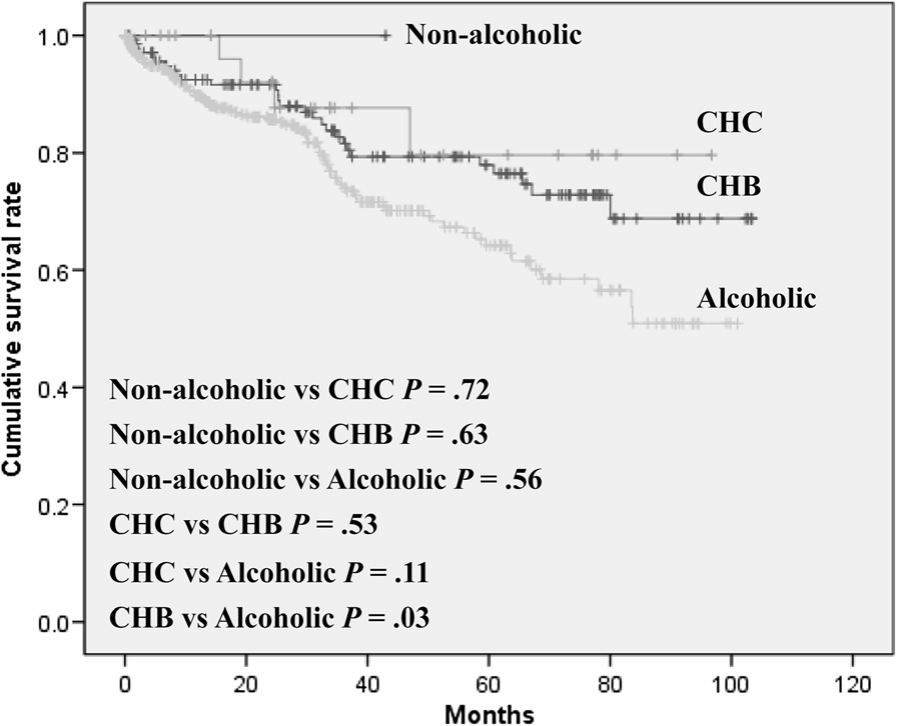
Survival curve according to the etiology of LC. LC, liver cirrhosis; HBV, hepatitis B virus; HCV, hepatitis C virus

The distribution of sex and etiology of LC was different between patients with PHG and without PHG. However, the survival rate was not different between men and women (*P* = 0.44, log-rank test). In terms of the etiology of LC, the survival rate was only different between patients with CHB-associated LC and those with alcoholic LC (*P* = 0.03) (Fig. 3). The development of HCC was not significantly different between men and women (*P* = 0.66, log-rank test). In terms of the etiology of LC, the survival rate was not also different according to the etiology (Additional file 1: Figure S1).

#### Multivariate analysis for the prediction of survival in patients with LC

In the multivariate analysis of independent risk factors for survival, PHG (severe PHG vs no PHG, HR: 3.29, 95 % CI: 1.12–9.63, *P* = 0.03) and Child-Pugh classification (Child C vs A, HR: 3.53, 95 % CI: 1.79–6.97, *P* < 0.001) (Child B vs A, HR: 2.15, 95 % CI: 1.35–3.44, *P* = 0.001) showed statistically significant associations with survival in patients with LC. Age (HR 1.03, 95 % CI: 1.01–1.06, *P* = 0.001) and HVPG (HR 1.06, 95 % CI: 1.03–1.08, *P* < 0.001) showed marginal statistical significance (Table 5). This analysis was controlled for age, sex, etiology of LC, and MELD score.

**Table 5:** Multivariable analysis for the prediction of survival in patients with LC

Variables, *n* (%)	HR (95 % CI)	*P* value
PHG	3.29 (1.12–9.63) (Severe PHG vs No PHG)	*P* = 0.03
Age	1.03 (1.01–1.06)	*P* = 0.001
Child-Pugh classification	3.53 (1.79–6.97) (Child C vs A)	*P* < 0.001
	2.15 (1.35–3.44) (Child B vs A)	*P* = 0.001
HVPG	1.06 (1.03–1.08)	*P* < 0.001

Controlled for age, sex, etiology, and MELD score

*N* number, *HVPG* hepatic vein pressure gradient, *PUD* peptic ulcer disease, *NSAIDs* non-steroid anti-inflammatory drugs, *LC* liver cirrhosis, *OR* Odds ratio

## Discussion

Blood flow congestion secondary to portal hypertension is considered the primary cause of PHG [17]. Imbalances between mucosal protective mechanisms and injury factors resulting from mucosal hemodynamic alterations are believed to induce PHG [18]. Although portal hypertension is the prerequisite for the development of PHG, various other factors, such as inflammatory response, local vascular tone, hepatic function, gastric mucosal perfusion, endotoxin, and gastric sucrose permeability, are suspected to influence the development of PHG [19–22]. Reversible mucosal changes in the stomach have suggested that PHG is a dynamic condition [23, 24]. Several studies have evaluated the correlation of PHG with the severity of liver disease or portal hypertension [5–13]. However, most of these studies have included small populations, and the association remains unclear. Our study included the largest population (*n* = 587) and evaluated the association of PHG with the development of HCC, as well as with portal hypertension and survival.

PHG was detected in 91.5 % of the total population in our study. This result was consistent with several previous studies (90.1 % in the study by Kim et al. and 93.4 % in the study by Curvêlo et al.) [7, 8]. The reported prevalence of PHG has shown great variation (7–98 %) [25, 26]. Selection bias in the studies, inconsistent endoscopic diagnosis criteria, and the lack of interobserver reliability are suspected causes of variation [27]. A recent study of the reliability of endoscopic diagnosis in PHG showed unsatisfactory results regarding the currently available diagnostic criteria (Baveno, McCormack, and NIEC classification) [27]. Binary criteria, such as the presence or absence of a mosaic-like pattern, red-point lesions and cherry-red spots, showed high inter-observer agreement and high specificity [27]. To minimize bias, our study adopted an analysis of group created according to the presence of PHG or not (binary criteria), in addition to the Baveno III scoring system for PHG, and six experienced endoscopists made decisions by consensus.

In the analysis of the correlation between PHG and the severity of liver disease, PHG showed correlations with Child-Pugh classification, HVPG, and MELD score, as well as laboratory parameters (Table 2). These correlations were also consistent in the analysis according to the severity of PHG (no PHG vs mild PHG vs severe PHG) (Table 3). This finding was consistent with a recent Korean study, which prospectively enrolled 331 patients with LC [8]. Previous studies indicating no definite correlation of PHG with the severity of liver disease have suggested that various factors are associated with the development of PHG [11, 12]. However, reversal or improvement of PHG was observed after treatment with transjugular intrahepatic portosystemic shunt (TIPS), indicating that portal hypertension is the main pathophysiologic mechanism of PHG [24]. These studies cannot definitely explain the cause of reversal of PHG after TIPS. Moreover, only small populations were included in these studies. Large scale studies, including our study, have shown a common association of PHG with the severity of liver disease [8].

Prognostic implications of PHG were also assessed in our study. The survival rates were statistically lower in patients with PHG, and this finding was consistent in the stratified analysis based on the severity of PHG (Tables 2 and 3; Fig. 2). Another large-scale study by Kim et al [8]. also showed consistent results. There was a report indicating an association of HVPG with mortality in patients with decompensated LC [28]. In this study, the cut-off value of HVPG of 18 mm Hg was associated with 2-year mortality in patients with decompensated LC [28]. This value was similar to our data of HVPG in patients with severe PHG (median HVPG 17.3 of mm Hg). Without definite mucosal hemorrhage, incidentally detected PHG is easily neglected by endoscopists. Although this study cannot provide histologic data about the liver, patients with PHG should be considered to have more advanced hepatic disease, associated with a poorer prognosis.

The presence of advanced hepatic fibrosis is related to portal hypertension and the development of HCC. Thus, early detection and treatment of liver fibrosis and its complications are important. After the suggestion of pathophysiologic classification of LC using HVPG by Garcia-Tsao et al. [29], the association of HVPG and the development of HCC have been studied. In a study of patients with decompensated alcoholic LC, the cut-off value for HVPG of 15 mm Hg was associated with the development of HCC [30]. However, in another study of patients with compensated LC, the cut-off value of HVPG 10 mm Hg was associated with the development of HCC [31]. According to our data, the presence of PHG could be used as an index of prognosis. However, the presence of PHG or the degree of PHG was not associated with the development of HCC. As shown in previous studies, many factors are associated with the development of HCC [32]. These factors include viral predisposing conditions, environmental factors, age, sex, genetic susceptibility, and metabolic factors [32]. Considering the risk persisting after sustained virological response in patients with HCV-associated LC or even after HBsAg seroclearance in patients with HBV infection, hemodynamic staging alone cannot predict the development of HCC [33–35].

There were several limitations of our study. This study lacked information about changes in PHG or HVPG according to the treatment of LC. The association of the development of PHG with esophageal or gastric varices could not be assessed because various treatments or even non-treated cases of varices were included in the total population. Despite these limitations, our study included the largest population, and long-term observation was undertaken for the evaluation of prognosis and the development of HCC.

## Conclusion

In conclusion, PHG was well correlated with portal hypertension. It could be used as a prognostic factor for LC but not for the prediction of HCC.

## Abbreviations

ALT, alanine transaminase; AST, aspartate transaminase; CI, confidence interval; EGD, esophagogastroduodenoscopy; HCC, hepatocellular carcinoma; HCV, hepatitis C virus; HR, hazard ratio; HVPG, hepatic vein pressure gradient; IQR, interquartile ranges; LC, liver cirrhosis; MELD score, The Model for End-stage Liver disease score; n, number; HBV, hepatitis B virus; PHG, portal hypertensive gastropathy

## Additional files

Additional file 1: Figure S1. Cumulative development of HCC curve according to the etiology of LC. HCC, hepatocellar carcinoma; LC, liver cirrhosis; HBV, hepatitis B virus; HCV, hepatitis C virus. (TIF 4549 kb)
