# Supplementary figures and images for: Portal hypertensive gastropathy as a prognostic index in patients with liver cirrhosis
Source: BMC Gastroenterol. 2016 Aug 12;16:93. doi: 10.1186/s12876-016-0508-2 (PMC4981996; doi:10.1186/s12876-016-0508-2)

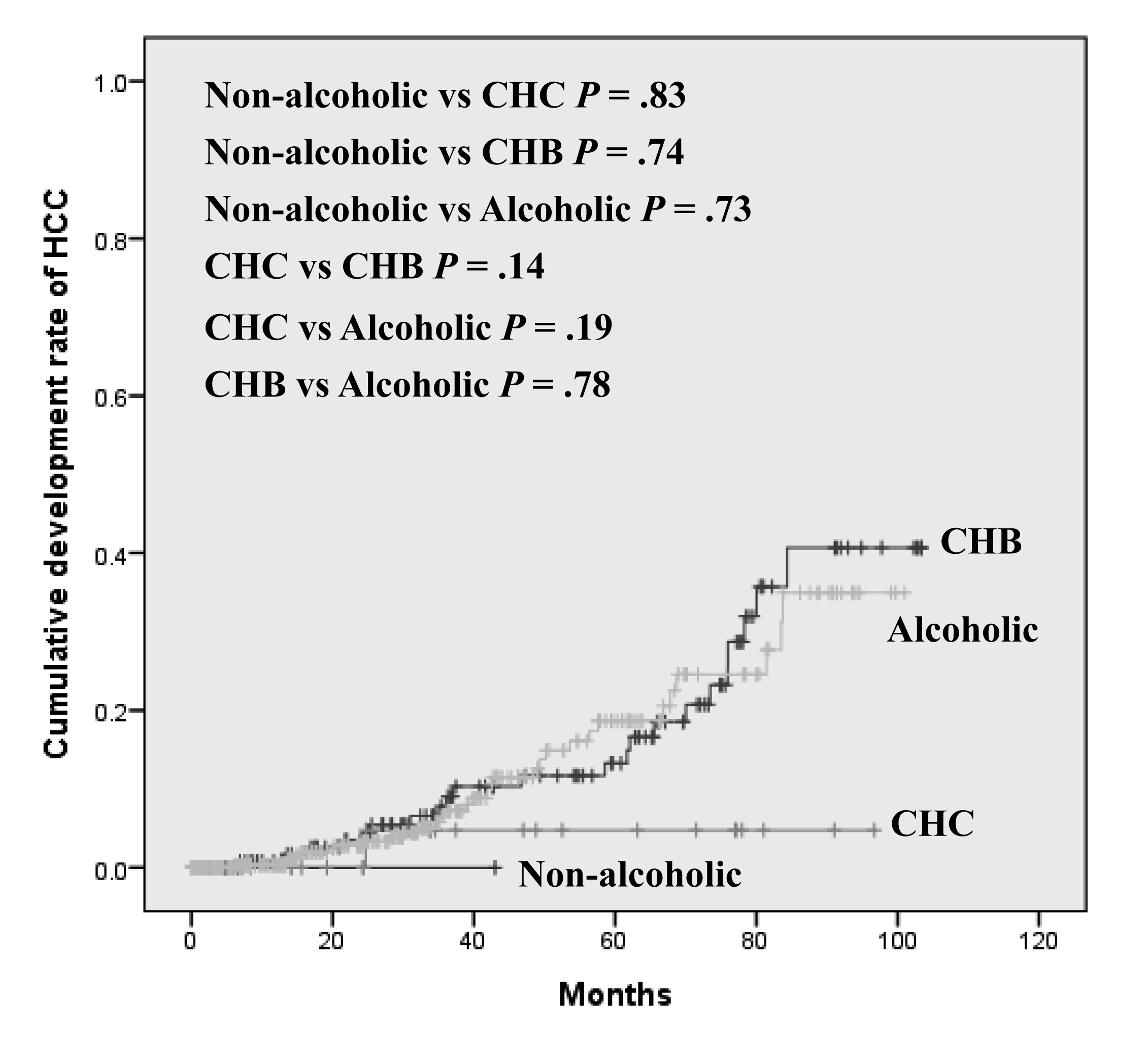

Supplement: Additional file 1: Figure S1. — Cumulative development of HCC curve according to the etiology of LC. HCC, hepatocellar carcinoma; LC, liver cirrhosis; HBV, hepatitis B virus; HCV, hepatitis C virus. (TIF 4549 kb) [file 12876_2016_508_MOESM1_ESM.tif]
